# Mitochondrial transfer in cancer: a global bibliometric analysis

**DOI:** 10.3389/fonc.2026.1872429

**Published:** 2026-07-08

**Authors:** Yang Lu, Liangkai Zhu, Yang Xu, Sihong Ye, Yana Wang, Baicheng Ni, Ming Zheng, Wanglong Chen

**Affiliations:** 1 Department of Anesthesiology, Yueqing Hospital of Wenzhou Medical University, Wenzhou, China; 2 Department of Emergency Medicine, Yueqing Hospital of Wenzhou Medical University, Wenzhou, China; 3 Clinical Medical College, Tianjin Medical University, Tianjin, China; 4 Department of Intensive Care Medicine, Yueqing Hospital of Wenzhou Medical University, Wenzhou, China

**Keywords:** bibliometric analysis, cancer therapy, mitochondrial transfers, mitochondrial transplantation, tumor microenvironment

## Abstract

**Background:**

Mitochondrial transfer is increasingly recognized as a biologically meaningful mode of intercellular communication in cancer. Its involvement in tumor metabolism, microenvironmental interaction, therapeutic adaptation, and immune regulation has driven rapid growth of the field. However, bibliometric assessment of its global research landscape has remained lacking.

**Methods:**

A bibliometric analysis was performed using literature retrieved from the Web of Science Core Collection, Scopus, and PubMed. English-language articles and reviews published between 1981 and 2025 were included. Bibliometrix, CiteSpace, and VOSviewer were used to evaluate publication trends, countries, institutions, authors, journals, citation networks, and keyword co-occurrence patterns.

**Results:**

A total of 184 publications were identified. Annual output increased sharply after 2016 and reached a peak of 48 papers in 2025. The literature was distributed across 117 journals, with 16 core journals identified according to Bradford’s law. The principal citation pathway extended from molecular, biology, and immunology journals to molecular, biology, and genetics journals, indicating a knowledge base rooted mainly in molecular and cellular mechanisms. China ranked first in publication output, followed by the United States. INSERM, Changhua Christian Hospital, and Sichuan University were the leading institutions. Neuzil J was the most productive author, whereas Berridge MV had the highest citation impact. The most cited reference was “Mitochondrial transfer between cells can rescue aerobic respiration”. Keyword evolution indicated a thematic shift from earlier emphases on mesenchymal stem cells, tunneling nanotubes, and mitochondrial transfer toward mitochondrial transplantation and the tumor microenvironment.

**Conclusions:**

Research on mitochondrial transfer in cancer has progressed from early mechanistic observation to a rapidly expanding field with increasing translational relevance. Current hotspots center on mitochondrial transplantation, tumor–microenvironment interactions, metabolic adaptation, and therapy-related biological processes such as chemoresistance and apoptosis. Further progress will require stronger causal validation, methodological standardization, and closer integration with clinically relevant models.

## 1 Introduction

Mitochondrial transfer is increasingly recognized as a non-canonical mode of intercellular communication, in which intact mitochondria or mitochondrial material are delivered from donor cells to recipient cells, thereby reshaping bioenergetic capacity, redox homeostasis, and cell fate (1, 2). A seminal observation was reported in 2006, when mtDNA-deficient A549 ρ° cells regained respiratory competence after acquiring mitochondria from neighboring cells in coculture, providing the first direct experimental foundation for intercellular mitochondrial transfer (3). Several transfer routes have since been characterized. Tunneling nanotubes are the most extensively studied and are widely regarded as the principal conduit for direct mitochondrial trafficking, particularly under stress conditions (1, 2, 4). Other mechanisms have also been reported, including extracellular vesicles, connexin-based gap junction channels, cell fusion, and uptake of extracellular mitochondria (2). Rather than representing a passive leakage phenomenon, mitochondrial transfer is increasingly viewed as stress-responsive and biologically regulated, and may be induced by hypoxia, oxidative injury, inflammation, or mitochondrial dysfunction in recipient cells (5). Its biological relevance has been explored in cancer, where experimental studies have suggested that transferred mitochondria may restore oxidative phosphorylation, increase ATP production, enhance metabolic plasticity, and support cell survival under selected therapeutic or microenvironmental stress conditions (6, 7).

Cancer has become a major setting for mitochondrial transfer research because malignant progression depends heavily on metabolic flexibility and microenvironmental support (8, 9). Mitochondria imported from stromal, endothelial, immune, or neighboring tumor cells can replenish oxidative phosphorylation, augment ATP production, and preserve biosynthetic capacity under stress (10, 11). In glioblastoma and hematologic malignancies models, this bioenergetic rescue has been linked to sustained proliferation, stem-like behavior, and greater tumorigenic fitness (12, 13). Beyond growth alone, mitochondrial transfer has also been associated with invasive and metastatic phenotypes, in part through restoration of respiratory function and in part through ROS-centered signaling effects (14). Evidence from breast cancer suggests that transferred mitochondria can buffer oxidative injury, compensate for OXPHOS-targeted stress, and facilitate escape from chemotherapy or endocrine therapy (15). As for tumor immunology, tumor cells may either appropriate mitochondria from cytotoxic lymphocytes or transmit dysfunctional mitochondria to T cells, resulting in metabolic exhaustion, impaired effector function, and reduced response to immune checkpoint blockade (10, 11). These findings place mitochondrial transfer at a biologically important intersection of tumor metabolism, intercellular crosstalk, therapeutic adaptation, and immune evasion, which explains its growing translational relevance in oncology.

Research on mitochondrial transfer in cancer has grown rapidly, and the field has become increasingly diverse in both theme and methodology. Recently studies have spanned metabolic reprogramming, microenvironmental support, immune regulation, therapy resistance, and translational exploration, with evidence emerging from multiple tumor types and experimental systems (1, 2). Bibliometric analysis provides a quantitative and visual approach for tracking publication growth, identifying major contributors, mapping collaborative networks, and revealing evolving hotspots (16–18). Bibliometric analysis may help delineate the knowledge structure and developmental trajectory of mitochondrial transfer research in cancer.

The present study aimed to systematically map the global research landscape of mitochondrial transfer in cancer through bibliometric analysis. This study examined publication trends, contributions by countries, institutions, authors, and journals, as well as citation networks and keyword co-occurrence patterns. The goal was to identify the field’s core knowledge base, major research hotspots, and shifts in research themes over time. These findings can help clarify the intellectual landscape of the field and offer useful guidance for future basic and translational research.

## 2 Materials and methods

### 2.1 Data source

For this study, relevant literature was systematically retrieved from three databases: Web of Science Core Collection (WoSCC), Scopus, and PubMed. All search operations were conducted on the same day to mitigate potential bias resulting from daily database updates. WoSCC, Scopus, and PubMed represent three major and widely utilized databases in the medical research domain. Both WoSCC and Scopus are multidisciplinary databases providing comprehensive coverage of scholarly publications, thereby offering researchers extensive literature support. In contrast, PubMed specializes in biomedical literature, delivering access to a substantial body of articles that may not be fully indexed in WoSCC or Scopus. The selection of these databases was aimed at ensuring that the bibliometric analysis draws upon a broad, authoritative, and diverse set of sources, thereby supporting a rigorous and comprehensive literature-based investigation.

### 2.2 Search strategy

A systematic literature search was conducted in Web of Science Core Collection, Scopus, and PubMed. The search strategy was designed around two concept groups. The first group included terms related to mitochondrial transfer, mitochondrial transplantation, mitochondrial trafficking, and mitochondrial exchange. The second group included terms related to cancer and neoplasms. The analysis was confined to articles and reviews published from 1981 to 2025, with the language restricted to English. Other types of literature were excluded from the analysis. As shown in **Supplementary Figure 1** and **Supplementary Table 2**, the overview of the bibliometric process was described.

The search identified 1,197 raw records, including 436 records from WoSCC, 456 records from Scopus, and 305 records from PubMed. Duplicate records were removed before eligibility screening. DOI, PubMed ID, and WoS accession number were used as strong matching identifiers. For records without DOI information or with inconsistent DOI information, titles were manually compared. When titles were identical or highly similar across databases, author information, publication year, and journal source were further checked. Two authors independently examined uncertain records using Microsoft Excel 2019, and disagreements were resolved through discussion. After deduplication, 620 unique records remained for screening. Two authors independently screened titles and abstracts according to predefined inclusion and exclusion criteria. Records were excluded if they were not related to mitochondrial transfer, mitochondrial transplantation, mitochondrial trafficking, mitochondrial exchange, or mitochondrial delivery in cancer. Because the final topic of this study was mitochondrial transfer or mitochondrial transplantation in cancer, excluded records were grouped into seven categories. The largest category included studies conducted in non-cancer disease settings, thematically irrelevant records, and records providing insufficient information to support inclusion. Other major exclusion categories included mitochondria-targeted drug delivery systems in cancer, cancer-related mitochondrial metabolism or function studies without mitochondrial transfer or transplantation, intracellular mitochondrial trafficking or protein translocation studies, methodological studies lacking a cancer-specific application, records related to mitochondrial transfer RNA rather than mitochondrial transfer, and broad reviews in which cancer or mitochondrial transfer was only briefly mentioned. The detailed exclusion categories are summarized in **Supplementary Table 3**.

### 2.3 Sensitivity check

To assess the potential influence of language restriction, we further performed a supplementary sensitivity check for non-English records (**Supplementary Table 4**). The same search strategy, publication period, and document-type criteria were applied in WoSCC, Scopus, and PubMed, but the English-language filter was removed. Non-English articles and reviews were then screened at the title and abstract level. After deduplication, 11 non-English records were retained, including 3 from WoSCC, 5 from PubMed, and 3 from Scopus. These records were not included in the main bibliometric network analysis because the formal analysis was designed based on a standardized English-language dataset. Instead, their titles, abstracts, languages, disease contexts, and major topics were manually reviewed to evaluate whether they introduced additional themes not captured by the main analysis.

### 2.4 Data standardization

To maintain terminological consistency and improve the accuracy of bibliometric mapping, data standardization was performed before formal analysis. Synonyms, spelling variants, singular and plural forms, and closely related expressions were unified by constructing a CiteSpace alias file and a VOSviewer thesaurus file. For keyword normalization, terms such as “mitochondria transfer,” “intercellular mitochondrial transfer,” and “intercellular transfer” were merged into “mitochondrial transfer.” Similar procedures were applied to other related terms, including mitochondrial transplantation, tunneling nanotubes, tumor microenvironment, oxidative phosphorylation, and mesenchymal stem cells when variant expressions were identified. Entity names were also standardized to reduce duplicated nodes in network visualization. Author name variants, institutional abbreviations, and country or region names were manually checked and unified when appropriate. For example, “United States” was standardized as “USA,” institutional abbreviations such as “CNRS” were unified with their full institutional names, and common variants of institutional names were merged after manual verification. These procedures were used to reduce artificial fragmentation in author, institution, country, and keyword networks. The merged terms and standardized entity names are provided in **Supplementary Tables 5**-**8**.

### 2.5 Software tools and respective functions

For the bibliometric analysis in this study, we utilized several established software tools, each chosen for its specific analytical strengths: the Bibliometrix R package, CiteSpace, and VOSviewer. The Bibliometrix R package was primarily used for quantitative analysis. In this study, its functions included counting publications and citations, evaluating the influence of source journals, analyzing keyword frequency and popularity, and assessing the intensity of international cooperation. CiteSpace was employed for citation analysis and visualization, aiding in the study of knowledge structure, distribution, and trends. Specifically, it was used to identify highly cited references and keywords within defined time periods. Additionally, VOSviewer served as our tool for co-authorship and co-occurrence analysis, enabling us to map collaborative networks among authors, their institutions, and across countries.

### 2.6 Clustering and visualization

VOSviewer (version 1.6.17) was employed to perform hierarchical clustering and visualize bibliometric data. As a widely recognized bibliometric analysis tool, VOSviewer enables the construction and interpretation of co-occurrence networks—such as keyword, author, institution, and country networks—to reveal thematic clusters and their interrelationships within a research domain. In this study, VOSviewer generated network visualizations that clearly delineate the intellectual structure, thematic evolution, and core subfields of mitochondrial transfer research. In the cluster analysis, the following screening criteria were used: keywords were included if they appeared at least five times in the dataset, countries were included if they had a minimum of 2 publications, organizations were included if they contributed at least 2 publications, and authors were included if they contributed at least two times. These thresholds were set to ensure that only the most important terms, countries, institutions, and authors were included in the analysis while minimizing the influence of rare terms.

## 3 Results

### 3.1 Quantitative analysis of publication

**Figure 1:**
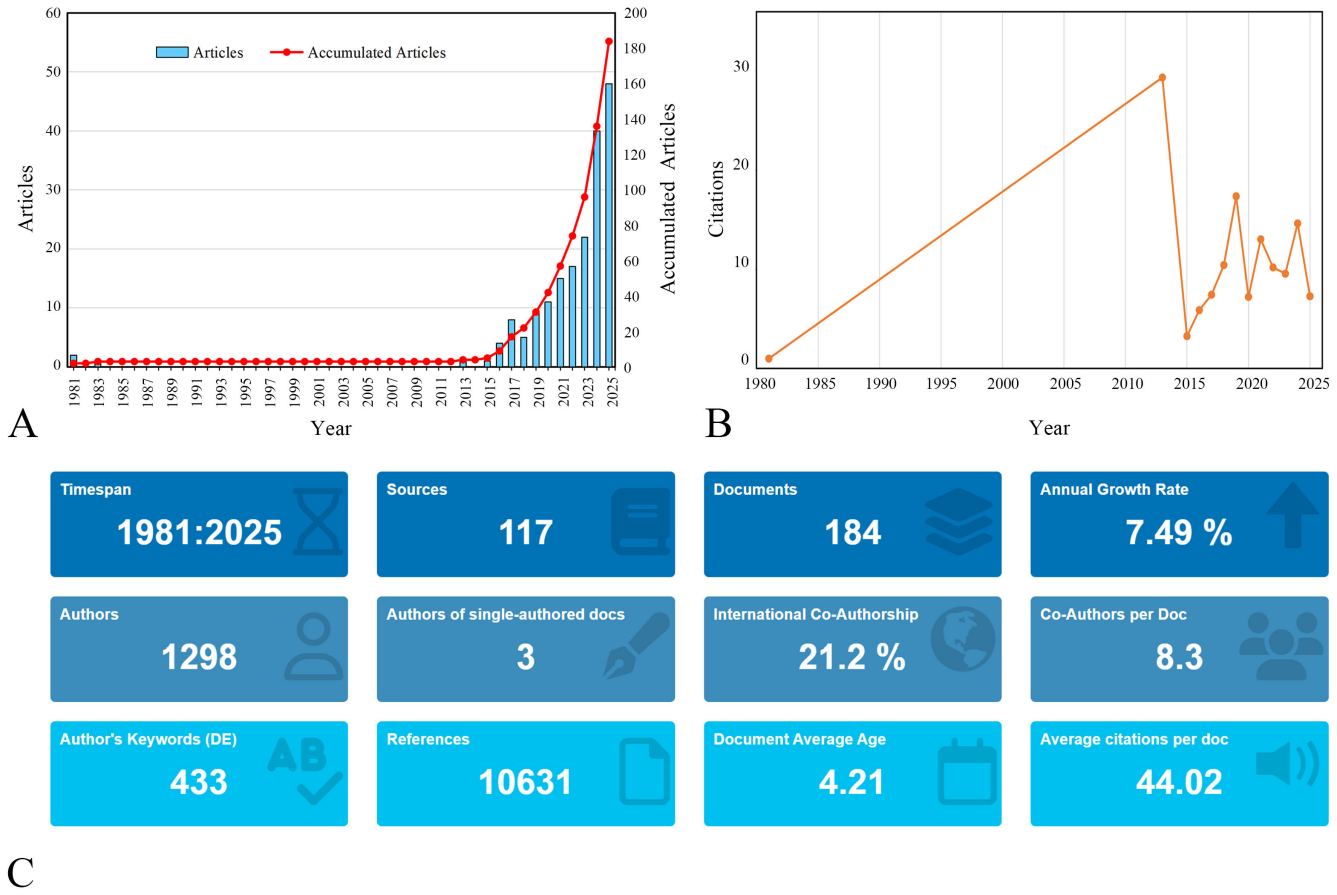
**(A)** The annual number and the cumulative number of publications. **(B)** The average annual citations of publications. **(C)** Additional statistics from R bibliometrix.

From January 1, 1981, to December 31, 2025, a total of 184 articles related to the field of mitochondrial transfer in cancer were retrieved (**Figure 1A**). The annual number of publications increased rapidly from 4 in 2016 to a peak of 48 in 2025. By 2025, the cumulative number of publications had reached 184. Analysis of the data from 1981 to 2025 revealed that the average citation rate per paper was 4.09 times per year. The highest peak occurred in 2013, with citations per article reaching 28.36 (**Figure 1B**). **Figure 1C** presents the reference information, journal sources, and relevant statistical data. In the **Supplementary Tables 4**, 11 non-English records were identified after deduplication and reviewed at the title and abstract level. These records included Chinese, French, Japanese, and Russian publications. The disease or research contexts included hematological malignancies, lung cancer, osteosarcoma, triple-negative breast cancer, tumor metabolism, mitochondrial medicine, and mitochondrial transplantation. The major topics identified from these records were consistent with the main bibliometric findings.

### 3.2 Analysis of journals

**Figure 2:**
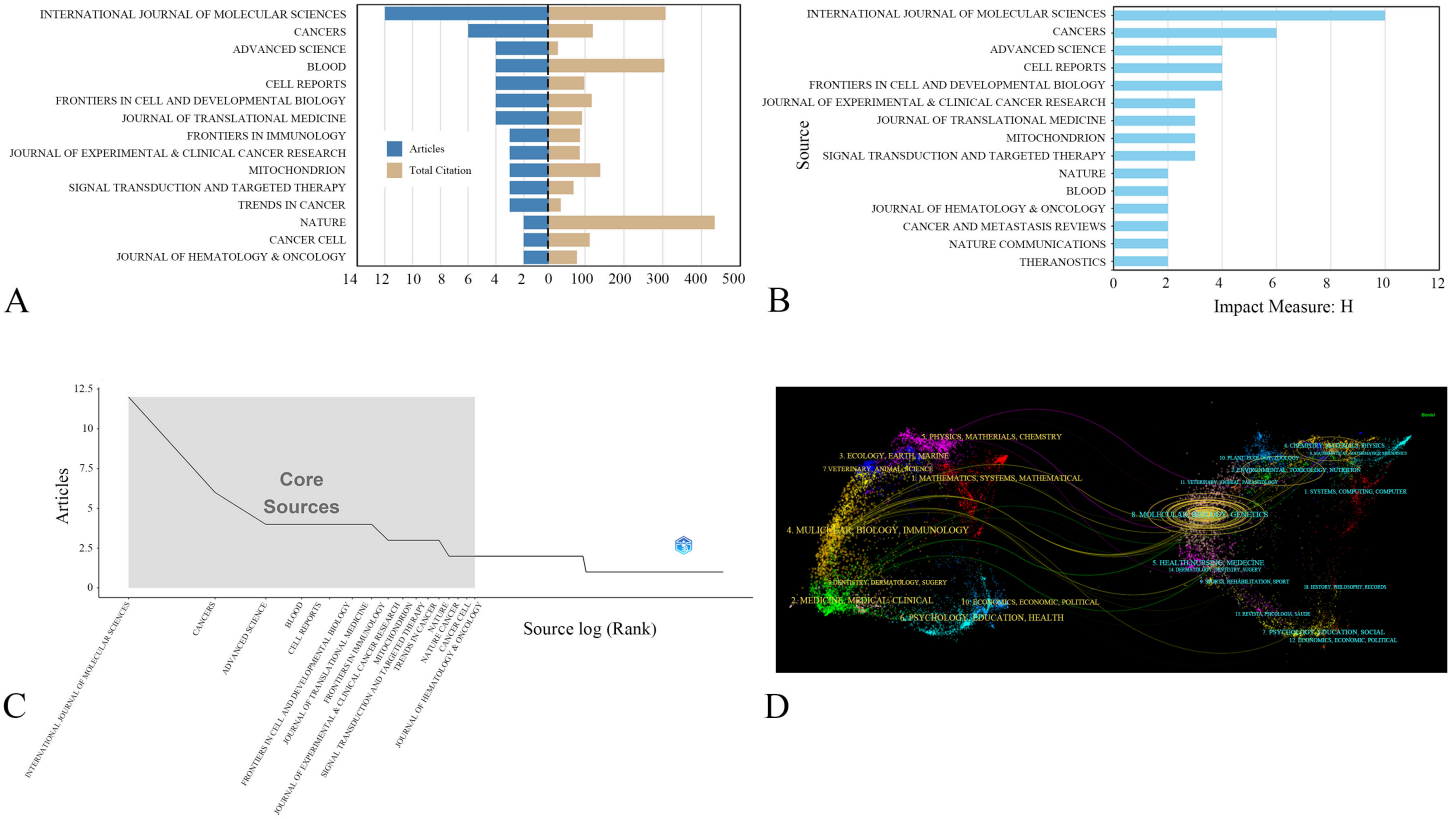
**(A)** The average citation and total publication number of the top 15 journals. **(B)** The top 15 Journals with the most H-index. **(C)** The delineation of core and non-core journals according to Bradford’s law. **(D)** Overlay graph of journal themes. The citing journals are located on the left, and the cited journals are located on the right.

In this study, publications were identified from a total of 117 journals. **Figure 2A** lists the 15 most productive journals in this field, along with their total number of publications and total citations. **Figure 2B** shows the top 15 journals based on the H-index, among which the *International Journal Of Molecular Sciences* ranks first. According to Bradford’s law, the 117 journals were categorized into 1–2 zones, with Zone 1 comprising 16 core source journals (**Figure 2C**). The Bradford core journals accounted for 61 publications from 16 sources, whereas Zone 2 and Zone 3 journals together contributed 123 publications from 101 sources. To visualize the relationship between citing and cited journals, we used a dual-map overlay of journals, as shown in **Figure 2D**. Different line colors represent different academic fields. One primary citation path was observed: a yellow line indicating that journals in the field of molecular/biology/immunology frequently cite journals in the field of molecular/biology/genetics.

### 3.3 Analysis of countries

**Figure 3:**
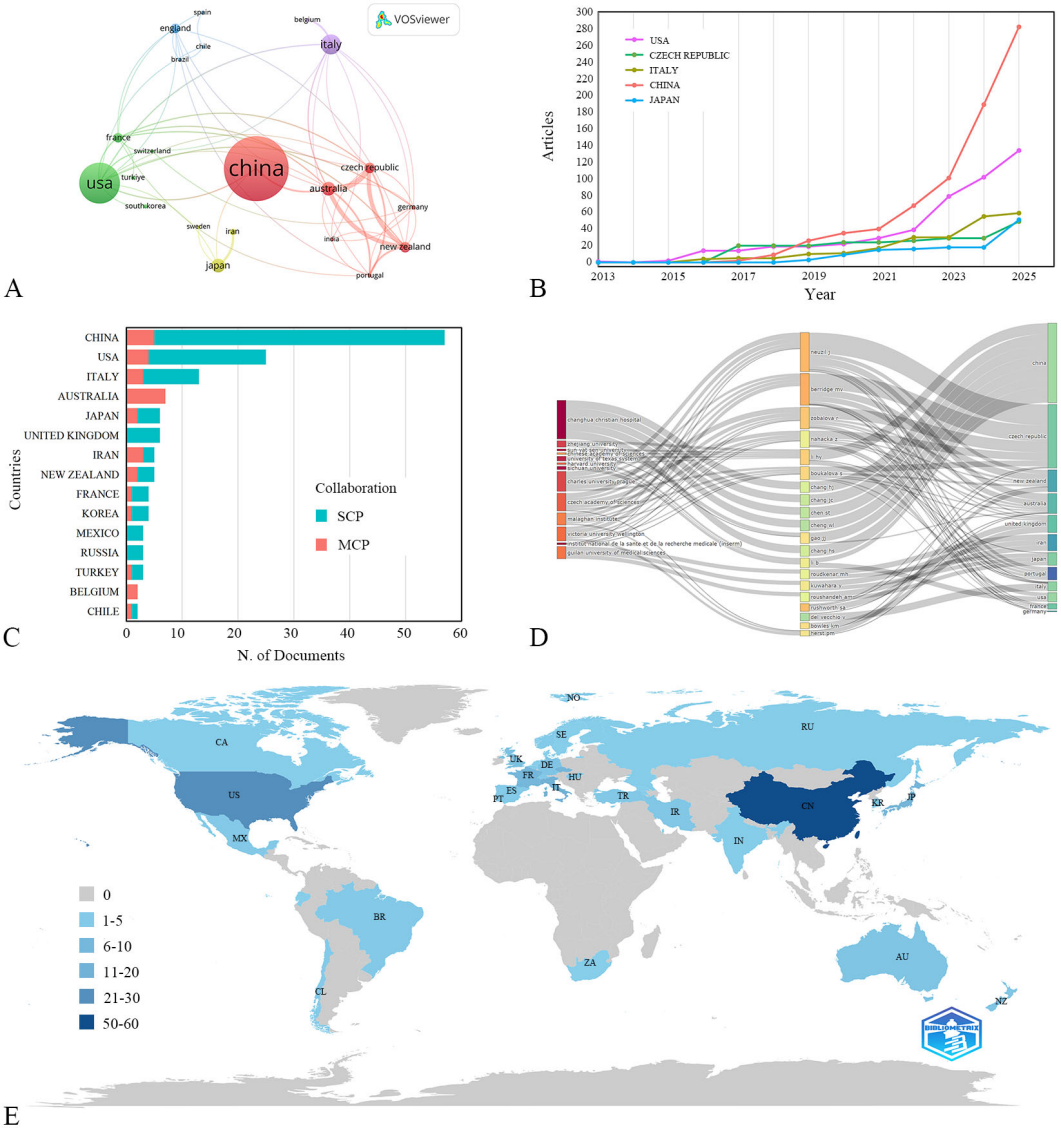
**(A)** Countries co-occurrence network. **(B)** Countries output trends within the top 5 from 1981 to 2025. **(C)** The proportion of cooperation between the top 15 countries with the most number of articles. **(D)** Three-field plot. **(E)** A map of country contribution based on the article output.

We then filtered and visualized data for the 35 countries that had at least one publication and constructed a collaboration network based on publication counts and the relationships between countries. As shown in **Figure 3A**, there is close cooperation among China, the United State, Australia, the New Zealand and European countries. The top 10 countries/regions ranked by the number of publications are listed in **Table 1**. Among them, the country with the largest contribution to publication is China (n= 57), followed by the United States (n=25). **Figure 3B** shows that annual publication output from the United States and China increased rapidly between 2015 and 2025. Most corresponding authors were affiliated with institutions in Australia (n=7, MCP = 100%) and China (n= 5, MCP = 8.8%), followed by the United States (n=4, MCP = 16%) (**Figure 3C**). Cross-links among the top 20 countries, institutions, and authors are shown (**Figure 3D**). The world map is presented in **Figure 3E**. The analysis of national publication output indicates that 35 countries have published articles in this field.

**Table 1:** The top 10 productive countries ranked by the number of publications.

Rank	Country	Articles	Citations	SCP	MCP
1	CHINA	57	1793	52	5
2	USA	25	1294	21	4
3	ITALY	13	668	10	3
4	AUSTRALIA	7	1358	0	7
5	JAPAN	6	396	4	2
6	UK	6	850	6	0
7	IRAN	5	126	2	3
8	NEW ZEALAND	5	159	3	2
9	FRANCE	4	80	3	1
10	KOREA	4	134	3	1

### 3.4 Analysis of institutions

**Figure 4:**
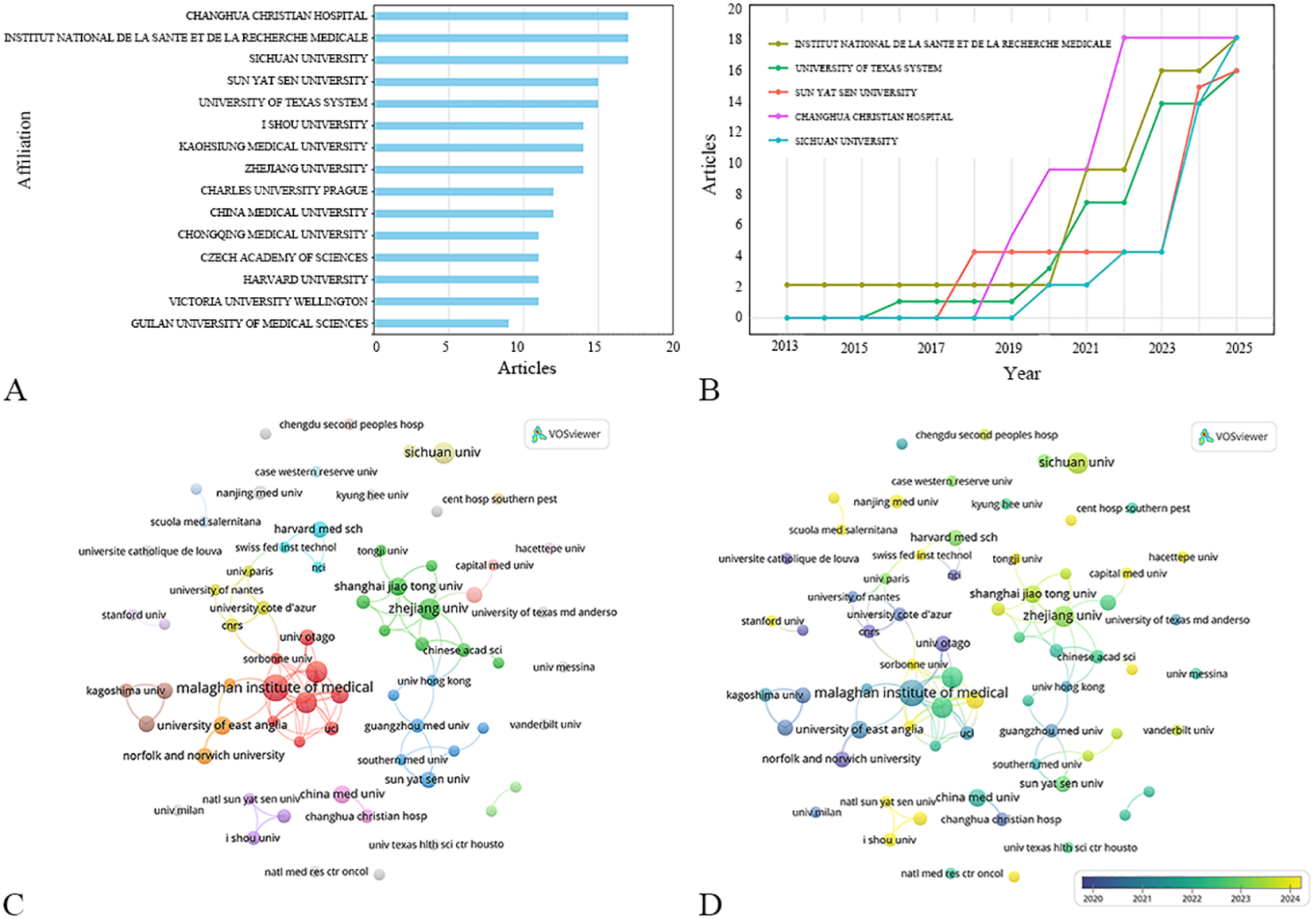
**(A)** The top 15 institutions with the most publications. **(B)** Institutions output trends within the top 5 from 1981 to 2025. **(C, D)** Institutions co-occurrence network.

A total of 344 institutions contributed to global scientific output. The top 15 institutions were summarized according to the number of publications (**Figure 4A**). Institut National de la Santé et de la Recherche Médicale (INSERM), Changhua Christian Hospital, and Sichuan University were ranked first with 17 papers. Among them, the production of Changhua Christian Hospital increased rapidly during the 2018–2022 period. By 2025, it remained one of the leading institutions, sharing the top position with INSERM and Sichuan University. (**Figure 4B**). To examine collaboration patterns, we constructed a network (**Figures 4C, D**) visualizing 69 institutions that had published at least two papers, with links representing both the scale of output and collaborative relationships between them.

### 3.5 Analysis of authors

**Figure 5:**
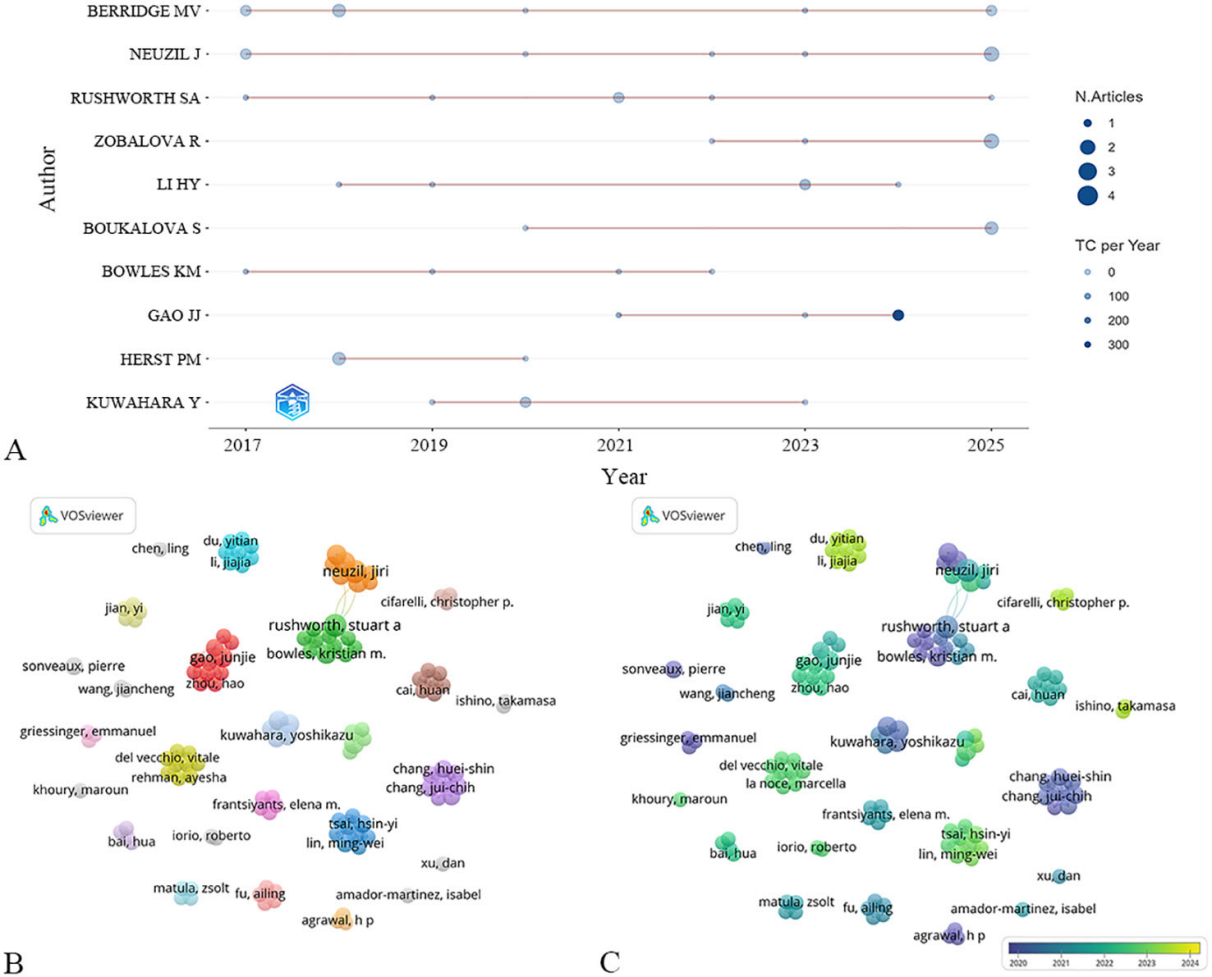
**(A)** Top authors’ production over the time. **(B, C)** Co-authorship network of the authors.

**Figure 5A** shows the publication output of leading authors over time. Analysis of author productivity reveals that from 2017 to 2025, the annual publication output of several leading scholars, including Berridge MV and Neuzil J, exhibited a fluctuating but overall upward trend. **Figure 5B** presents the collaboration network among 124 authors, each with more than two publications. The network comprises 26 clusters. According to the superimposed plots, Neuzil J and Rushworth SA worked closely together in this area. However, the author collaboration network reveals that international and inter-institutional cooperation among global authors remained relatively limited (**Figure 5C**). Analysis of author distribution showed that Berridge MV had the highest number of publications (8 articles, 239 citations), while GAO JJ received the most citations (9 articles, 1266 citations). The top 10 authors significantly influence research on mitochondrial transfer in cancer, with more than 4000 citations and a high H-index (**Table 2**).

**Table 2:** The top 10 productive authors ranked by the number of publications.

Rank	Author	Documents	Citations	H-index
1	BERRIDGE MV	9	239	7
2	NEUZIL J	9	214	7
3	RUSHWORTH SA	6	578	4
4	ZOBALOVA R	6	169	5
5	LI HY	5	559	5
6	BOUKALOVA S	4	53	3
7	BOWLES KM	4	578	4
8	GAO JJ	4	1266	4
9	HERST PM	4	124	3
10	KUWAHARA Y	4	202	4

### 3.6 Reference with citation bursts

**Figure 6:**
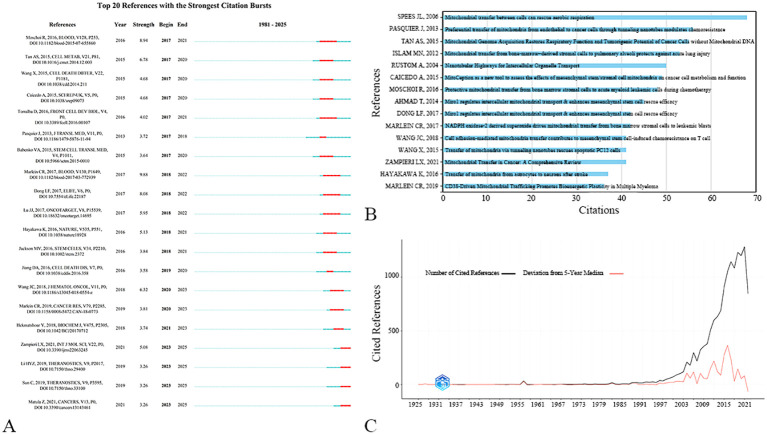
**(A)** The top 20 references with the strongest citation bursts. **(B)** The top 15 references with the most local citations. **(C)** Reference publication year spectroscopy.

A citation burst denotes a publication that receives a sharp, temporary increase in citations within a specific field. According to our analysis using CiteSpace, a total of 20 articles showed strong citation bursts (**Figure 6A**). **Supplementary Table 1** lists the top 10 most frequently cited articles, Spees JL published in the journal *Proc Natl Acad Sci U S A* entitled “Mitochondrial transfer between cells can rescue aerobic respiration “ was the most cited article (n=901). The top 15 most cited references are presented in **Figure 6B**. The citation trajectories for the top three locally cited references show that their combined citation count increased rapidly from 2007, peaking in 2021 (**Figure 6C**).

### 3.7 Keyword visualization and bursts

**Figure 7:**
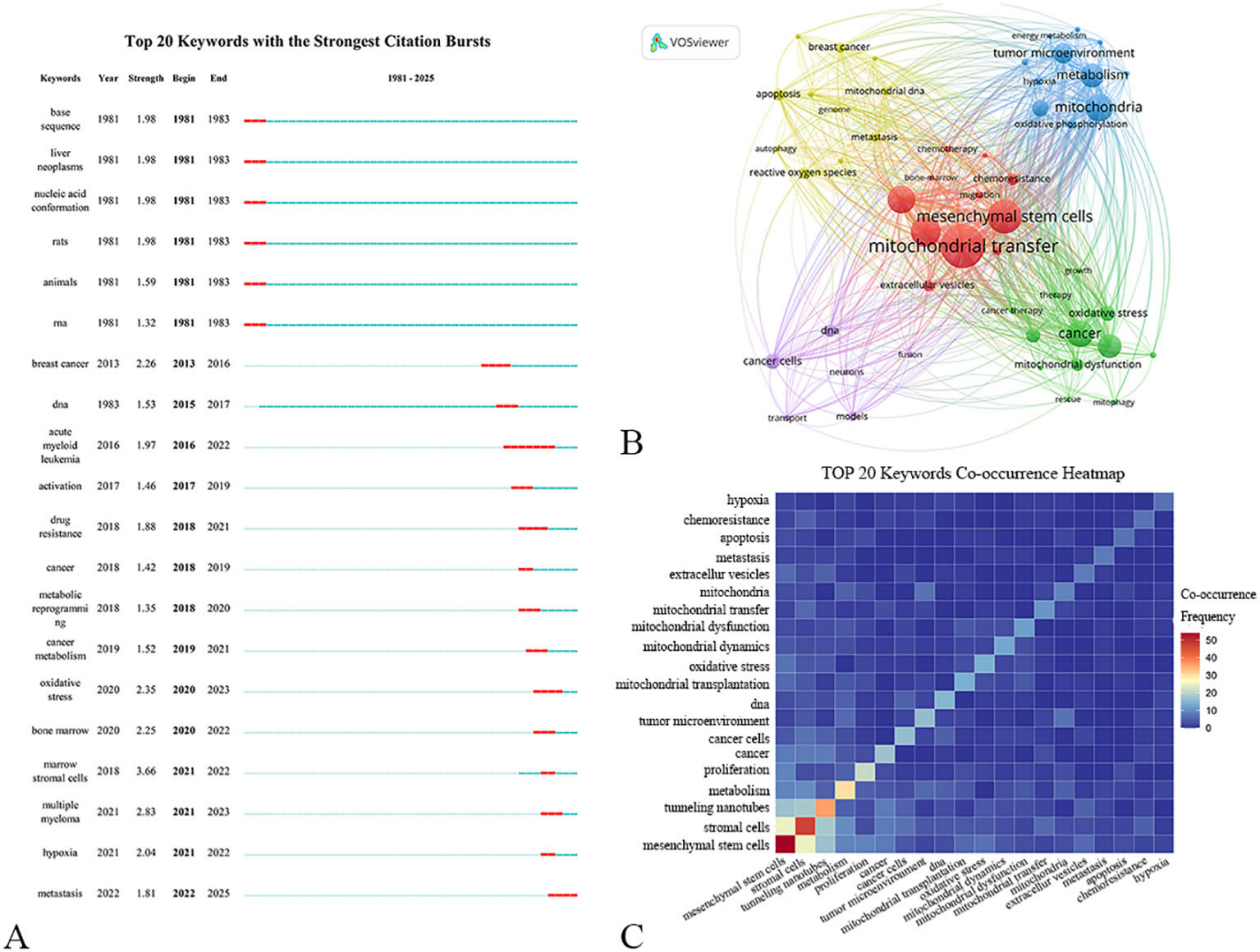
**(A)** The top 20 keywords with the strongest citation bursts. **(B)** Keyword cluster analysis. **(C)** Analysis of co-occurrence heat map of the top 20 keywords.

**Figure 7A** presents the top 20 keywords with the strongest citation bursts. “acute myeloid leukemia” (2016–2022, 7 years) was the most concerned keywords over time. Furthermore, more recent keywords such as “bone marrow”, “marrow stromal cells”, “multiple myeloma”, “hypoxia”, and “metastasis” (2020-2025) have emerged as prominent, indicating current and potential future research hotspots. As shown in **Figure 7B**, keywords with frequency greater than 5 are given. A total of 51 high-frequency keywords were identified and grouped into 5 clusters. **Figure 7C** shows the co-occurrence frequency of the top 20 keywords, with warmer colors (red/orange/yellow) indicating higher co-occurrence intensity. The dark-red link between “mesenchymal stem cells” and “stromal cells” signifies their strong association and highlights this as a predominant research focus. Similarly, yellow links connecting “tunneling nanotubes” with “metabolism”, suggest that these terms have been frequently discussed together in the literature. Keywords such as “hypoxia”, “chemoresistance”, and “apoptosis” mainly show low frequency co-occurrence with other terms. This pattern suggests they represent relatively independent research streams that have not yet converged deeply with core thematic modules.

**Figure 8:**
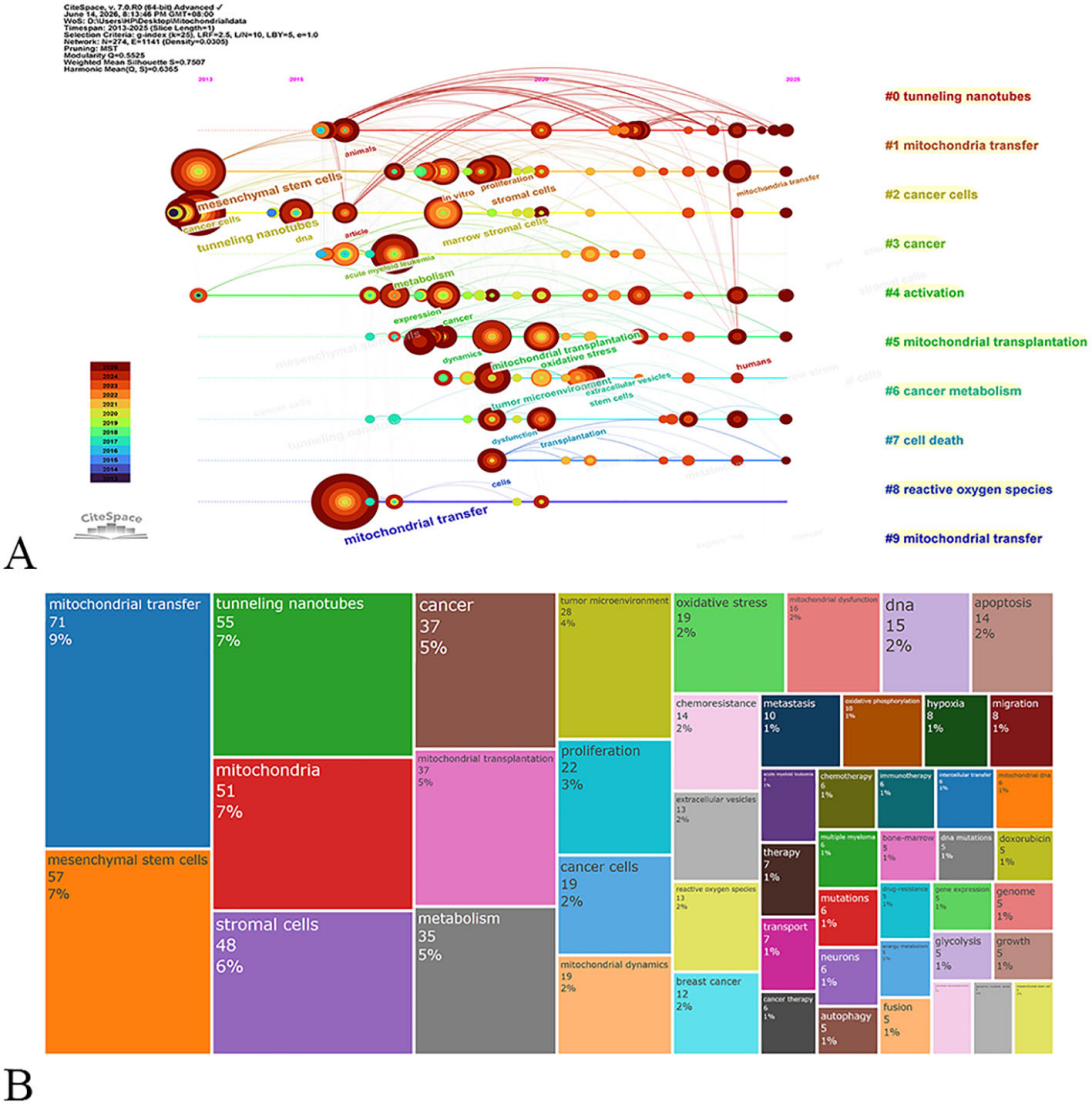
**(A)** Clustering analysis of keywords timeline viewer on mitochondrial transfer in cancer. **(B)** Word tree representation of the top 50 keyword occurrences.

Using the log-likelihood ratio (LLR) algorithm, we categorized all identified keywords into ten clusters. **Figure 8A** illustrates the temporal development and thematic evolution of keywords within each cluster. From 2013 to 2016, the main keywords were “mesenchymal stem cell”, “tunneling nanotubes” and “mitochondrial transfer”. From 2017 to 2025, the focus shifted to terms such as “mitochondrial transplantation” and “tumor microenvironment”. This analysis helps to track the evolution of specific topics within the field over time and to identify emerging developments and frontiers. The keyword “mitochondrial transfer” appeared most frequently (n= 71, 9%), This was followed by “mesenchymal stem cells” (n= 57, 7%), “tunneling nanotubes” (n=55, 7%), “mitochondria” (n= 51, 7%) and “stromal cells” (n= 48, 6%) (**Figure 8B**).

## 4 Discussion

In the present study, bibliometric analysis was performed to characterize the global research landscape of mitochondrial transfer in cancer. The United States and China were the leading contributors, and annual publication output increased markedly after 2016, reaching a peak in 2025. Journal analysis showed that the literature was distributed across 117 journals, with the main citation pathway centered on molecular biology and genetics. Author analysis further indicated that representative scholars, particularly Neuzil J and Berridge MV, have made substantial contributions to this field, although broader collaboration among research groups remained limited. Overall, mitochondrial transfer in cancer appears to be a relatively young but rapidly evolving research field, characterized by a growing publication base, a concentrated academic core, and increasing literature attention to tumor biology-related topics.

### 4.1 Global distribution of research power and collaboration pattern

The top ten countries in terms of publication output on mitochondrial transfer in cancer were China, the United States, Italy, Australia, Japan, England, Iran, New Zealand, France, and Korea (**Table 1**). The geographical distribution of publications showed a clear concentration in countries with relatively strong biomedical research capacity and established scientific infrastructure. This pattern is broadly consistent with the technical demands of mitochondrial transfer research, which often relies on advanced experimental approaches, including live-cell imaging (19), co-culture systems (20), organelle tracing (21), and metabolic functional assays (22). This distribution may be partly associated with differences in research infrastructure, technical capacity, and the visibility of biomedical publications. Although China ranked first in publication output, the United States accumulated a substantially higher citation count, indicating that publication volume and academic influence were not fully aligned in this field. This finding suggests that, despite the rapid expansion of Chinese research, the United States may still retain an advantage in high-impact and innovation-driven studies, particularly in research involving emerging technologies and novel directions. This interpretation is also supported by the field’s early intellectual foundation. A seminal study published in 2006 showed that mtDNA-deficient A549 ρo cells regained respiratory competence after acquiring mitochondria from neighboring cells, providing the first direct experimental basis for intercellular mitochondrial transfer (3). In the present bibliometric analysis, the study by Spees JL, Mitochondrial transfer between cells can rescue aerobic respiration, was also identified as the most cited reference in the field. Such an advantage may be related, at least in part, to sustained investment in scientific research, stronger innovation infrastructure, and earlier deployment of advanced biomedical research platforms (2, 23).

A distinct pattern of international collaboration was also observed. The collaboration network indicated close cooperation among China, the United States, Australia, New Zealand, and several European countries. Some countries with relatively smaller publication outputs also showed active cross-national links, suggesting that publication productivity and collaborative engagement were not entirely overlapping dimensions. Notably, Australia showed a high proportion of multiple-country publications, indicating its prominent role in international collaboration within this research field. Countries with stronger funding support, more mature biomedical platforms, and more established foundational research systems have been more likely to occupy leading positions in this field, whereas developing countries may still face constraints related to limited investment and weaker research capacity (24, 25). Under such circumstances, closer collaboration with leading research countries may help strengthen academic development and improve participation in emerging areas of investigation (26). At the same time, future changes in funding priorities and the broader geopolitical environment may still influence patterns of international cooperation, particularly among major contributors, and could therefore affect the subsequent development of the field (27, 28). Overall, research on mitochondrial transfer in cancer remains concentrated in a relatively small number of high-capacity countries, while broader multinational collaboration has yet to be fully established.

### 4.2 Analysis of research categories

The dual-map overlay of journals showed that the principal citation pathway extended from molecular/biology/immunology to molecular/biology/genetics (**Figure 7**). Based on citation patterns, the knowledge base of mitochondrial transfer research in cancer appears to be mainly associated with molecular biology, immunology, genetics, mitochondrial functional regulation, and intercellular communication, rather than with large-scale clinical investigation or standardized therapeutic research (2, 6). This interpretation was further reinforced by the distribution of institutions and authors. Existing studies have been driven largely by a limited number of highly active institutions and representative investigators (5, 29). INSERM, Sichuan University and Changhua Christian Hospital were ranked first in publication output. These findings indicate that progress in this field has depended, to a considerable extent, on core research centers with established strengths in mitochondrial biology, cancer research, and translational experimental platforms (30, 31). At the author level, Neuzil J ranked highest in publication output, whereas Berridge MV showed greater citation impact. Together with Rushworth SA, they formed a relatively close collaborative cluster. This pattern of sustained output from a small number of core teams suggests that advances in the current research categories have relied less on a widely distributed academic community and more on relatively concentrated mechanism-oriented research forces (6, 32, 33). The Bradford journal distribution also provides useful insight into the structure of this field. The core journals mainly reflect the stable thematic backbone of research on mitochondrial transfer in cancer, including mitochondrial transfer, tunneling nanotubes, the tumor microenvironment, stromal cells, mesenchymal stem cells, oxidative phosphorylation, and therapy resistance. However, journals outside the Bradford core also contributed several distinctive and emerging topics. In particular, studies published in lower-output or peripheral journals extended the field toward immune-cell mitochondrial hijacking, platelet-mediated metabolic crosstalk, mitochondrial transplantation-based radiosensitization, and imaging-based single-mitochondrion analysis (11, 34–36). These findings suggest that peripheral journals should not be regarded as marginal in scientific value. Instead, they may serve as an exploratory space where specialized or potentially field-shaping concepts first appear before being further consolidated in higher-output journals. Overall, core journals define the recurrent research backbone of the field, whereas peripheral journals broaden its conceptual and methodological boundaries.

The current research profile can therefore be described as mechanism-oriented at the literature level, with frequent attention to topics related to tumor metabolic adaptation, intercellular communication, and microenvironmental interaction. This interpretation reflects the disciplinary orientation of the literature and should not be considered direct mechanistic validation by the present bibliometric study. The field is beginning to extend toward translational application and broader interdisciplinary development.

### 4.3 Overview of knowledge base and research hotspots

Research on mitochondrial transfer in cancer has evolved from a loose and weakly connected collection of mitochondrial observations into a more coherent and rapidly expanding field. The earliest studies were concerned mainly with mitochondrial components within tumor cells rather than with intercellular mitochondrial trafficking in the modern sense (37, 38). A genuine conceptual shift emerged in 2006, when it was demonstrated that mitochondria could be transferred between cells and restore respiratory function in recipient cells. This changed the perspective of the field. Mitochondria were no longer viewed only as intracellular therapeutic targets, but increasingly as transferable organelles with functional consequences (3, 39). Between 2010 and 2016, the field entered an early phase of mechanistic expansion. During this period, the potential routes of mitochondrial transfer were gradually delineated. These included extracellular vesicle-associated transport of mitochondrial components and intercellular exchange mediated by tunneling nanotubes (40, 41). It should be noted that these routes are not biologically equivalent. Tunneling nanotubes are generally associated with direct cell-to-cell transfer of relatively intact mitochondria, whereas extracellular vesicles may carry intact mitochondria, mitochondrial fragments, mtDNA, mitochondrial proteins, or other mitochondria-derived cargos. At the same time, the biological context widened. Attention moved beyond intracellular mitochondrial biology alone and began to incorporate tumor–stromal interactions and early translational exploration (42–44). After 2016, greater attention was given to the functional consequences of mitochondrial transfer in specific tumor settings. Several experimental studies within the cited literature have reported that bone marrow stromal cells may alleviate oxidative stress in leukemic cells and support their survival through mitochondrial transfer. These studies provide biological context for the observed research themes but do not represent causal conclusions derived from the present bibliometric analysis (7, 32). In bladder cancer, tunneling nanotube-mediated mitochondrial transfer has been reported to be associated with enhanced invasiveness (45). In prostate cancer and multiple myeloma, studies have linked mitochondrial transfer mediated by cancer-associated fibroblasts or bone marrow stromal cells to metabolic rewiring, bioenergetic plasticity, and therapeutic tolerance (46, 47). Around 2020 to 2021, research on mitochondrial transfer began to enter what may be termed the tumor microenvironment era (48, 49). The literature was no longer satisfied with describing the transfer phenomenon itself. More emphasis was placed on its specific effects on tumor behavior (50). A study suggested that mitochondrial transfer could contribute to glioblastoma adaptation to hypoxia and metabolic stress (51). Mitochondrial transfer could also mediate energetic support from stromal cells to tumor cells and was associated with drug resistance in diseases such as multiple myeloma (52). The key change during this period was that mitochondrial transfer began to move beyond being regarded as a mechanistic phenomenon and became an increasingly important perspective for understanding adaptive tumor survival and treatment response (50). From 2022 onward, mitochondrial transplantation (53), tumor microenvironment remodeling (54, 55), multidrug resistance (15), and immune regulation (56, 57) gradually moved to the forefront. This suggests that the field has progressed beyond early route identification and has become increasingly concerned with biological consequences and therapeutic exploitability. Publications on mitochondrial transfer in cancer remained limited before 2016 but increased rapidly thereafter. The keyword profile also shifted from early terms such as “mesenchymal stem cell” and “tunneling nanotubes” toward later terms such as “mitochondrial transplantation,” and “tumor microenvironment.” This keyword pattern indicates that recent publications have increasingly focused on topics related to metabolic adaptation, tumor–microenvironment interaction, therapeutic resistance, and immune regulation. The emergence of immune regulation as a recent research hotspot highlights the need to interpret mitochondrial transfer within the broader framework of T-cell metabolic exhaustion and immune dysfunction (58). Antitumor T-cell activity depends on mitochondrial fitness, oxidative phosphorylation, redox balance, and metabolic flexibility. However, chronic antigen stimulation, hypoxia, nutrient depletion, lactate accumulation, oxidative stress, and inhibitory receptor signaling within the tumor microenvironment can impair mitochondrial function and promote T-cell exhaustion. Scharping et al. showed that the tumor microenvironment represses T-cell mitochondrial biogenesis and drives intratumoral T-cell metabolic insufficiency and dysfunction (59). While Liu et al. further demonstrated that hypoxia can induce mitochondrial defects associated with T-cell exhaustion (60). In this context, Ikeda et al. provided important evidence that mitochondrial transfer contributes to immune evasion by altering the metabolic state of T cells (11). Therefore, tumor-related mitochondrial transfer may serve as a mechanistic link between cancer metabolic reprogramming, T-cell metabolic exhaustion, and impaired antitumor immunity.

### 4.4 Future directions and translational challenge

Bibliometric evidence suggests that the focus of this field has shifted from earlier themes such as mesenchymal stem cells, tunneling nanotubes, and mitochondrial transfer toward mitochondrial transplantation and the tumor microenvironment, with hypoxia and metastasis emerging as recent research hotspots. Future work is likely to converge on three major priorities. The relative importance of distinct transfer routes across tumor entities and microenvironmental settings needs to be delineated (49). From a biological and translational perspective, future experimental studies will be needed to define whether and how mitochondrial transfer contributes to metabolic adaptation, proliferative fitness, invasive behavior, metastatic competence, and therapeutic resistance (61, 62). Just as importantly, it remains to be established whether this process can be translated into a tractable therapeutic target in oncology (63).

Keyword co-occurrence patterns further reveal strong links between mesenchymal stem cells and tunneling nanotubes, between cancer cells and membrane nanotubes, and between mitochondrial transfer and oxidative stress. These associations point to donor–recipient heterogeneity within the tumor microenvironment as a major unresolved issue (10). The relative contribution of stromal, immune, and endothelial compartments to mitochondrial donation remains poorly resolved. Another question is more fundamental: is mitochondrial transfer merely a transient stress-buffering response, or does it act as a durable mechanism that reprograms tumor cell state and reshapes the surrounding niche? This distinction matters (61). From a therapeutic perspective, several avenues deserve attention, including blockade of tumor-promoting mitochondrial transfer, interference with tunneling nanotube- or extracellular vesicle-mediated delivery, and evaluation of mitochondrial transplantation in selected oncologic settings (53). Progress in these areas will also depend on greater multicenter coordination, more rigorous technical standardization, and broader use of live-cell tracing, single-cell omics, spatial transcriptomics, and integrative multi-omics approaches (57, 64). More comparable datasets and more reproducible experimental pipelines will be essential if the field is to move from mechanistic interpretation to functional validation and eventual translational application.

Despite its clear mechanistic interest and translational promise, this field still faces several major obstacles. No consensus has yet been reached on the relative contribution of different transfer modes across tumor types, donor–recipient pairs, and stress states (49). Much of the available evidence still comes from *in vitro* co-culture systems or selected animal models (1). High-resolution, dynamic validation of mitochondrial origin, trafficking, and functional consequences within the native *in vivo* tumor microenvironment remains limited (48). Increasing links have been drawn between mitochondrial transfer and metabolic rewiring, stromal support, therapy resistance, and immune escape (11). Whether these relationships are causally instructive, however, or simply reflect adaptive phenomena under tumor stress, remains unsettled. Additional translational barriers are also evident. Donor–recipient specificity, delivery efficiency *in vivo*, long-term safety, and the definition of appropriate clinical indications all require careful resolution before meaningful clinical application can be envisioned (53, 65). Conceptual standardization is needed. Future studies should clearly specify whether the observed event represents transfer of intact mitochondria, transfer of mitochondria-containing vesicles, or transfer of mitochondria-derived materials such as mtDNA, mitochondrial proteins, or mitochondrial fragments (66). Methodological standardization is also needed because the detection of mitochondrial transfer remains technically challenging. Imaging-based approaches and mitochondrial dyes are widely used, but fluorescence signals may be affected by dye leakage, non-specific staining, photoconversion-related artifacts, or transfer of mitochondrial fragments rather than intact functional mitochondria (67). Therefore, future studies should not rely on a single dye-based or fluorescence-based assay. Genetically encoded mitochondrial reporters, donor–recipient cell-specific labeling, live-cell imaging, three-dimensional reconstruction, mtDNA or mitochondrial haplotype tracing, electron microscopy, and functional assays of respiration or ATP production should be combined to provide stronger evidence for genuine mitochondrial transfer (29).

## 5 Limitation

This study has several limitations. Database bias cannot be completely avoided. Although three major databases were searched, incomplete database coverage and differences in indexing may still have led to omission of relevant studies. In addition, only English-language articles and reviews were included. Although we performed an additional title-and-abstract-level sensitivity check of non-English records retrieved from WoSCC, Scopus, and PubMed using the same search strategy, these records were not incorporated into the formal bibliometric network analysis. Language-related selection bias cannot be completely excluded. Bibliometric results are also influenced by the search strategy and term normalization process, and some studies using alternative terminology may not have been captured. Although the search strategy included the core term “mitochondrial transfer” and several closely related mitochondrial-specific expressions, some relevant studies using broader mechanistic terminology, such as “organelle transfer,” or “tunneling nanotubes,” may not have been fully captured. Moreover, citation bias should be considered when interpreting the results. Citation-based indicators tend to favor older publications, whereas recent studies, particularly those published close to the retrieval date, may have been underestimated. Finally, bibliometric analysis can reveal structural trends and thematic evolution, but it cannot directly evaluate the methodological quality or causal strength of the underlying studies. Therefore, we were unable to classify every included article according to whether it examined intact mitochondrial transfer or component-level mitochondrial transfer.

## 6 Conclusion

This bibliometric analysis delineates the global structure and developmental trajectory of research on mitochondrial transfer in cancer. The field has expanded rapidly since 2016, with publication activity concentrated in a limited number of countries, institutions, and research groups. Its knowledge base remains rooted primarily in molecular and cellular mechanisms, while recent thematic evolution indicates increasing attention to mitochondrial transplantation, tumor microenvironmental interaction, therapeutic resistance, and immune regulation. These patterns suggest that mitochondrial transfer has moved beyond a descriptive biological phenomenon and is becoming an important framework for understanding tumor adaptation. However, the field remains constrained by limited causal evidence, incomplete translational validation, and insufficient large-scale integration across research systems. Further progress will depend on more rigorous mechanistic studies, improved methodological standardization, and closer alignment with clinically relevant models.

## Data Availability

The data analyzed in this study is subject to the following licenses/restrictions: The datasets used and/or analyzed during the current study are available from the corresponding author on reasonable request. Requests to access these datasets should be directed to Wanglong Chen, cwl830112@163.com.

## Supplementary material

Supplementary Figure 1 Research flowchart of literature searching and screening.

Supplementary Table 1 The top 10 papers ranked by the number of citations.

Supplementary Table 2 Search strategies used in different databases.

Supplementary Table 3 Articles were excluded from inclusion criteria.

Supplementary Table 4 Non-English records identified in the title-and-abstract-level sensitivity check.

Supplementary Table 5 Combined by the same authors.

Supplementary Table 6 Combined by the same countries.

Supplementary Table 7 Combined by the same institutions.

Supplementary Table 8 Combined by the same keywords.
