# Supplementary material for: Mitochondrial transfer in cancer: a global bibliometric analysis
Source: Front Oncol. 2026 Jul 8;16:1872429. doi: 10.3389/fonc.2026.1872429 (PMC13388204; doi:10.3389/fonc.2026.1872429)
Supplement: Supplementary Figure 1 — Research flowchart of literature searching and screening. [file DataSheet1.docx]

**Figure S1**. Research flowchart of literature searching and screening.


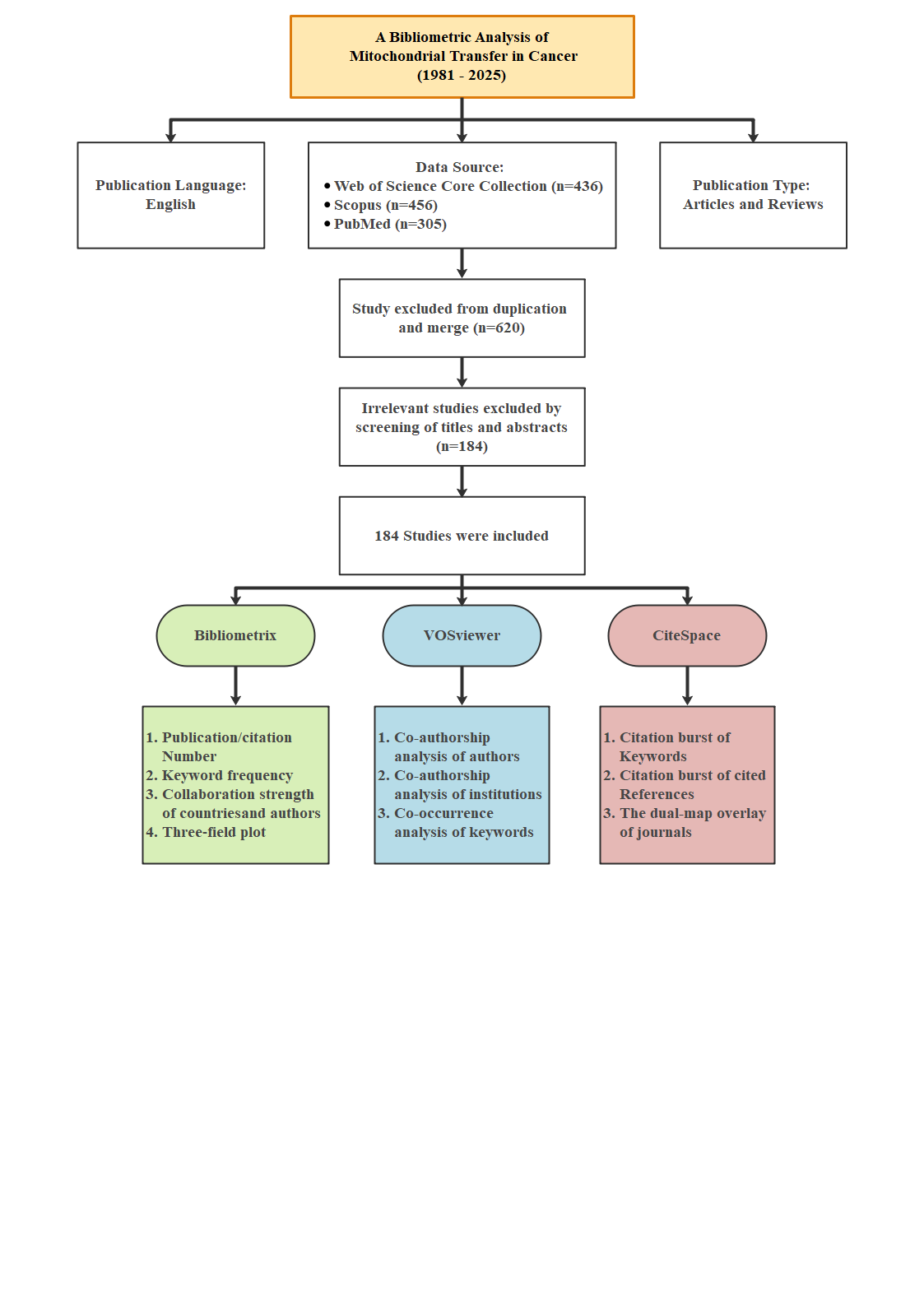


Supplementary Table 1. Top 10 papers ranked by the number of citations

| **Rank** | **Year** | **Source** | **Title** | **IF** | **JCR** | **Citations** |
| --- | --- | --- | --- | --- | --- | --- |
| 1 | 2006 | Proc Natl Acad Sci USA | [Mitochondrial transfer between cells can rescue aerobic respiration](https://www.webofscience.com/wos/woscc/full-record/WOS:000235094300025) | 9.1 | Q1 | 901 |
| 2 | 2013 | J Transl Med | [Preferential transfer of mitochondria from endothelial to cancer cells through tunneling nanotubes modulates chemoresistance](https://www.webofscience.com/wos/woscc/full-record/WOS:000319897400001) | 7.5 | Q1 | 397 |
| 3 | 2015 | Cell  Metab | Mitochondrial Genome Acquisition Restores Respiratory Function and Tumorigenic Potential of Cancer Cells without Mitochondrial DNA | 30.9 | Q1 | 630 |
| 4 | 2012 | Nat Med | Mitochondrial transfer from bone-marrow-derived stromal cells to pulmonary alveoli protects against acute lung injury | 50 | Q1 | 1268 |
| 5 | 2004 | Science | Nanotubular highways for intercellular organelle transport | 45.8 | Q1 | 1612 |
| 6 | 2015 | Sci Rep | MitoCeption as a new tool to assess the effects of mesenchymal stem/stromal cell mitochondria on cancer cell metabolism and function | 3.9 | Q1 | 282 |
| 7 | 2016 | Blood | Protective mitochondrial transfer from bone marrow stromal cells to acute myeloid leukemic cells during chemotherapy | 23.1 | Q1 | 368 |
| 8 | 2014 | EMBO J | Miro1 regulates intercellular mitochondrial transport & enhances mesenchymal stem cell rescue efficacy | 8.3 | Q1 | 516 |
| 9 | 2017 | Elife | Horizontal transfer of whole mitochondria restores tumorigenic potential in mitochondria! DNA-deficient cancer cells | 5.4 | Q1 | 216 |
| 10 | 2017 | Blood | NADPH oxidase-2 derived superoxide drives mitochondrial transfer from bone marrow stromal cells to leukemic blasts | 23.1 | Q1 | 283 |

**Supplementary Table 2.** **Search strategies used in different databases**

| **Database** | **Search field** | **Mitochondrial movement-related terms** | **Cancer-related terms** | **Limits and filters** |
| --- | --- | --- | --- | --- |
| Web of Science Core Collection (436) | Topic search, TS | “Mitochondrial transfer” OR “Mitochondrial transplantation” OR “Mitochondrial migration” OR “Mitochondrial intercellular transport” OR “Mitochondrial delivery” OR “Mitochondrial trafficking” OR “Mitochondrial exchange” | “cancer” OR “tumors” OR “neoplasia” OR “neoplasm” OR “tumor” OR “malignant neoplasm” OR “malignancy” OR “malignancies” OR “benign neoplasm” | Document type: Article or Review; Publication years: 1981–2025 |
| Scopus  (456) | Title, abstract, and keywords, TITLE-ABS-KEY | “Mitochondrial transfer” OR “Mitochondrial transplantation” OR “Mitochondrial migration” OR “Mitochondrial intercellular transport” OR “Mitochondrial delivery” OR “Mitochondrial trafficking” OR “Mitochondrial exchange” | “cancer” OR “tumors” OR “neoplasia” OR “neoplasm” OR “tumor” OR “malignant neoplasm” OR “malignancy” OR “malignancies” OR “benign neoplasm” | Document type: Article or Review; Language: English; Publication years: 1981–2025 |
| PubMed (305) | Title and abstract, [tiab] | “Mitochondrial transfer” OR “Mitochondrial transplantation” OR “Mitochondrial migration” OR “Mitochondrial intercellular transport” OR “Mitochondrial delivery” OR “Mitochondrial trafficking” OR “Mitochondrial exchange” | “cancer” OR “tumors” OR “neoplasia” OR “neoplasm” OR “tumor” OR “malignant neoplasm” OR “malignancy” OR “malignancies” OR “benign neoplasm” | Language: English; Publication date: January 1, 1981 to December 31, 2025; Publication type: Journal Article or Review; Excluded publication types: Case Reports, Editorials, Comments, Letters, and News |

**Supplementary Table 3.** Articles were excluded from inclusion criteria

| Reasons for exclusion | Quantity | Percentage | Explanation |
| --- | --- | --- | --- |
| Mitochondrial transfer/transplantation in the context of non-neoplastic disease | 177 | 40.4% | The main subjects of research are cardiovascular disease, nerve injury, spinal cord injury, kidney disease, hepatic ischemia-reperfusion, pulmonary fibrosis, sarcopenia, inflammatory diseases, etc. Although mitochondrial transfer or mitochondrial transplantation was present, it was not a neoplastic background. |
| Mitochondrial targeted delivery systems in the context of cancer | 101 | 23.2% | Mainly include mitochondria-targeted nanoparticles, liposomes, TPP modification, DOX delivery, mitochondrial-targeted nanodrugs, etc. This belongs to mitochondrial-targeted therapy, not mitochondrial transfer or transplantation. |
| Study on mitochondrial metabolism/function of tumors, but did not involve mitochondrial transfer/transplantation | 67 | 15.4% | The main topics discussed include OXPHOS, mtDNA, ROS, apoptosis, mitochondrial dynamics, mitochondrial quality control, and tumor metabolic reprogramming, but there was no mention of intercellular mitochondrial transfer or mitochondrial transplantation. |
| The transport, localization or protein translocation of mitochondria within the cell is not the transfer between cells. | 52 | 12% | For instance, mitochondrial trafficking, mitochondrial migration, mitochondrial translocation, p53/Bcl2 translocation to the mitochondria, etc. These are all intracellular processes and do not involve the transfer of mitochondria from one cell to another. |
| Methodological or technical platform articles lack clear applications for tumors. | 37 | 8.5% | The main methods include mitochondrial injection, mitoception, mitochondrial separation/delivery, robot-assisted mitochondrial injection, cybrid technology, etc. These can serve as background information for the methods, but they are not related to tumor application research. |
| The general review only briefly mentions tumor or mitochondrial transfer. | 2 | 0.5% | The topic is rather broad. Cancer or mitochondrial transfer are just brief mentions within the context of numerous diseases. |

Supplementary Table 4. Non-English records identified in the title-and-abstract-level sensitivity check

| No. | Database source | First author | Year | Title | Language | Cancer or disease context | Main topic identified at title/abstract level |
| --- | --- | --- | --- | --- | --- | --- | --- |
| 1 | WoSCC | Yamada Y | 2012 | Targeting Mitochondria: Innovation from Mitochondrial Drug Delivery System (DDS) to Mitochondrial Medicine | Japanese | Mitochondrial diseases, cancer, and neurodegenerative disorders | Mitochondrial drug delivery system, mitochondrial medicine, mitochondrial gene therapy |
| 2 | WoSCC | Yang SF | 2018 | Advances of Mitochondrial Transplantation Therapy for The Mitochondrial Deficiency Diseases | Chinese | Mitochondrial deficiency diseases, with discussion of tumor therapy | Mitochondrial transplantation, mitochondrial dysfunction, cerebral ischemia, myocardial ischemia-reperfusion injury, and tumor-related therapeutic potential |
| 3 | WoSCC | Boët E | 2024 | Mitohormesis: the keystone of therapeutic resistance of cancer cells | French | Acute myeloid leukemia and cancer therapy resistance | Mitochondrial metabolism, mitohormesis, oncometabolism, therapeutic resistance |
| 4 | PubMed | Huang NW | 2021 | Research progress in mitochondrial transfer mediated by tunneling nanotube in the field of tumor | Chinese | Tumor | TNT-mediated mitochondrial transfer, mitochondrial respiratory deficiency, metabolic reprogramming, therapeutic targets |
| 5 | PubMed | Zhang LY | 2022 | Research Progress of Intercellular Mitochondrial Transfer in the Development of Hematological Malignant Tumors --Review | Chinese | Hematological malignancies | Intercellular mitochondrial transfer, bone marrow mesenchymal stem cells, leukemia, multiple myeloma, tunneling nanotubes, chemotherapy resistance |
| 6 | PubMed | Ma LL | 2024 | Effects of normal mitochondrial transplantation on proliferation, apoptosis and stemness of triple-negative breast cancer cells | Chinese | Triple-negative breast cancer | Normal mitochondrial transplantation, proliferation, apoptosis, stemness, breast cancer cell phenotype |
| 7 | PubMed | Li YQ | 2025 | Effects of mitochondrial transplantation on full-thickness skin defects in diabetic rats | Chinese | Diabetic wound model, not cancer-specific | Mitochondrial transplantation, oxidative stress, mitochondrial membrane potential, wound healing |
| 8 | Scopus | Yamada Y | 2017 | MITO-Porter, multifunctional envelope-type nano device for mitochondrial delivery toward innovative nano medicine | Japanese | Mitochondrial diseases, including cancer-related mitochondrial dysfunction | Mitochondrial delivery, nano medicine, mitochondrial drug delivery systems |
| 9 | Scopus | Wu F | 2024 | Research Progress in Mitochondrial Treatment and Mechanism in Occurrence of Lung Cancer | Chinese | Lung cancer | Mitochondrial-targeted drugs, mitochondrial transfer, mitochondrial gene therapy, lung cancer treatment |
| 10 | Scopus | Qin Q | 2025 | Mitochondrial Transfer Promotes Immune Escape in Osteosarcoma Cells: Mechanisms and Research Advances | Chinese | Osteosarcoma | Mitochondrial transfer, immune escape, metabolic regulation, oxidative stress, immune checkpoint regulation, tumor microenvironment |
| 11 | Scopus | Lin T | 2025 | Research progress and application prospects of platelet-derived mitochondrial transfer | Chinese | Multiple diseases, including cancer | Platelet-derived mitochondrial transfer, cell communication, bioenergetics, metabolic reprogramming, therapeutic applications |

Note: These non-English records were identified after removing the language restriction and were reviewed at the title and abstract level only. They were not incorporated into the main bibliometric network analysis, but were used to assess whether language restriction may have excluded major conceptual, experimental, or regional themes. The main bibliometric dataset remained restricted to English-language articles and reviews.

**Supplementary Table 5. Combined by the same authors**

| **Label** | **Replace by** |
| --- | --- |
| berridge, michael v. | berridge, michael, v |
| herst, p. m. | herst, patries m |
| rushworth, stuart a. | rushworth, stuart a |

**Supplementary Table 6. Combined by the same countries**

| **Label** | **Replace By** |
| --- | --- |
| Peoples r China | China |
| Taiwan | China |
| Scotland | England |
| United States | USA |

**Supplementary Table 7. Combined by the same institutions**

| **Label** | **Replace By** |
| --- | --- |
| Acad Sci Czech Republ | Czech Academy Of Sciences |
| Czech Acad Sci | Czech Academy Of Sciences |
| Centre National De La Recherche Scientifique | Cnrs |
| Ctr Natl Rech Sci Cnrs Gdr3697 | Cnrs |
| Fau Erlangen Nurnberg | Friedrich Alexander University Erlangen-Nurnberg |
| Friedrich Alexander Univ Fau | Friedrich Alexander University Erlangen-Nurnberg |
| Aou Luigi Vanvitelli | University Of Campania Luigi Vanvitelli |
| Univ Campania l Vanvitelli | University Of Campania Luigi Vanvitelli |
| Univ Campania Luigi Vanvitelli Via Leonardo Bianch | University Of Campania Luigi Vanvitelli |
| Malaghan Inst Med Res | Malaghan Institute Of Medical Research |
| Univ Cote Azur | University Cote d'Azur |
| Univ Cote Azur Uca | University Cote d'Azur |
| Université Côte d'Azur | University Cote d'Azur |
| Univ Nantes | University Of Nantes |
| Université De Nantes | University Of Nantes |
| Kaohsiung Med Univ Hosp | Kaohsiung Medical University |
| Okayama Univ Hosp | Okayama University |
| Jilin Univ | Jilin University |
| First Hosp Jilin Univ | Jilin University |
| Houston Methodist Hosp | Houston Methodist |
| Houston Methodist Res Inst | Houston Methodist |
| Tours Univ | University Of Tours |
| Tours Univ Hosp | University Of Tours |
| Univ Montpellier | University Of Montpellier |
| Univ Hosp Ctr Montpellier Brc | University Of Montpellier |
| Chiba Canc Ctr | Chiba Cancer Center |
| Chiba Canc Ctr Res Inst | Chiba Cancer Center |
| Norfolk & Norwich Univ Hosp | Norfolk And Norwich University Hospital Nhs Trust |
| Norfolk & Norwich Univ Hosp Nhs Trust | Norfolk And Norwich University Hospital |
|  | Nhs Trust |
| Univ Ostrava | University Of Ostrava |
| Univ Hosp Ostrava | University Of Ostrava |
| Univ Regensburg | University Of Regensburg |
| Univ Hosp Regensburg | University Of Regensburg |
| Univ Aquila | University Of l'Aquila |
| Univ Catholique Louvain Uclouvain | Universite Catholique De Louvain |
| Catholic Univ Louvain | Universite Catholique De Louvain |
| Kaohsiung Med Univ | Kaohsiung Medical University |
| Natl Inst Cardiol Ignacio Chavez | Instituto Nacional De Cardiologia Ignacio Chavez |
| Inst Nacl Cardiol Ignacio Chavez | Instituto Nacional De Cardiologia Ignacio Chavez |
| Univ Nacl Autonoma Mexico | National Autonomous University Of Mexico |
| Natl Autonomous Univ Mexico Unam | National Autonomous University Of Mexico |
| Md Anderson | University Of Texas Md Anderson Cancer Center |
| Univ Texas Md Anderson Canc Ctr | University Of Texas Md Anderson Cancer Center |
| Tohoku Med & Pharmaceut Univ | Tohoku Medical And Pharmaceutical University |
| Univ East Anglia | University Of East Anglia |
| Norwich Medical School | University Of East Anglia |

**Supplementary Table 8. Combined by the same keywords**

| **Label** | **Replace By** |
| --- | --- |
| mesenchymal stem-cells | mesenchymal stem cells |
| membrane nanotubes | tunneling nanotubes |
| cancer-cells | cancer cells |
| cell proliferation | proliferation |
| mitochondrion | mitochondria |
| ros | reactive oxygen species |
| breast-cancer | breast cancer |
| marrow stromal cells | stromal cells |
| transplantation | mitochondrial transplantation |
| dynamics | mitochondrial dynamics |
| dysfunction | mitochondrial dysfunction |
| stem-cells | mesenchymal stem cells |
| microenvironment | tumor microenvironment |
| tnt formation | tunneling nanotubes |
| resistance | chemoresistance |
| intercellular mitochondrial transfer | mitochondrial transfer |
| intercellular transfer | mitochondrial transfer |
| mesenchymal stem cell | mesenchymal stem cells |
| multiple-myeloma | multiple myeloma |
| mtdna | mitochondrial dna |
| leukemia, myeloid, acute | acute myeloid leukemia |
| multiple-myeloma | multiple myeloma |
| mtdna | mitochondrial dna |
| mesenchymal stem cell | mesenchymal stem cells |
| mitochondria transfer | mitochondrial transfer |
| intercellular transfer | mitochondrial transfer |
| intercellular mitochondrial transfer | mitochondrial transfer |
| tumor | cancer |
| model | models |
